# UHPLC–Q/Orbitrap/MS/MS Fingerprinting, Free Radical Scavenging, and Antimicrobial Activity of *Tessaria absinthiodes* (Hook. & Arn.) DC. (Asteraceae) Lyophilized Decoction from Argentina and Chile

**DOI:** 10.3390/antiox8120593

**Published:** 2019-11-28

**Authors:** Jessica Gómez, Mario J. Simirgiotis, Beatriz Lima, Carlos Gamarra-Luques, Jorge Bórquez, Duilio Caballero, Gabriela Egly Feresin, Alejandro Tapia

**Affiliations:** 1 Instituto de Biotecnología-Instituto de Ciencias Básicas, Universidad Nacional de San Juan, Av. Libertador General San Martín 1109 (O), San Juan CP 5400, Argentina; jesicagomez674@gmail.com (J.G.); blima@unsj.edu.ar (B.L.); gferesin@unsj.edu.ar (G.E.F.); 2 CONICET (Consejo Nacional de Ciencia y Tecnología), CABA, Buenos Aires C1405DJR, Argentina; cgamarraluques@gmail.com; 3 Instituto de Farmacia, Facultad de Ciencias, Universidad Austral de Chile, Campus Isla Teja, Valdivia 5090000, Chile; 4 Center for Interdisciplinary Studies on the Nervous System (CISNe), Universidad Austral de Chile, Valdivia 5090000, Chile; 5 Instituto de Medicina y Biología Experimental de Cuyo, CONICET-Universidad Nacional de Cuyo, Mendoza CP5500, Argentina; 6 Facultad de Ciencias Médicas, Universidad Nacional de Cuyo, Mendoza CP5500, Argentina; 7 Laboratorio de Productos Naturales Depto. de Química, Facultad de Ciencias, Universidad de Antofagasta, Av. Coloso S-N, Antofagasta 1240000, Chile; jorge.borquez@uantof.cl; 8 Laboratorio Hospital Marcial Quiroga, Av. Libertador General San Martín 5401 (O), Rivadavia, San Juan CP 5407, Argentina; duiliocaballero@gmail.com

**Keywords:** UHPLC–Q-Exactive focus, sesquiterpenes, phenolics acids, flavonoids, antioxidant

## Abstract

The decoction of *Tessaria absinthioides* is used in traditional medicine of South America as hypocholesterolemic, balsamic, and expectorant; but it is also useful for the prevention of hepatitis, renal insufficiency, and diabetes, and is used as digestive. A lyophilized decoction from the aerial parts of this plant (TLD) collected in San Juan (TLDSJ) and Mendoza (TLDM) provinces (Argentina) and one collection from Antofagasta, Chile (TLDCH) were characterized regarding antioxidant and antibacterial activities, phenolics and flavonoids content, and ultrahigh resolution liquid chromatography Orbitrap MS analysis UHPLC–PDA–OT-MS/MS metabolite profiling. The antioxidant properties were carried out "in vitro" using 2,2-diphenyl-1-picrylhydrazyl (DPPH) and trolox equivalent antioxidant activity (TEAC) methods, ferric-reducing antioxidant power (FRAP), and lipoperoxidation in erythrocytes (LP). The antibacterial activity was evaluated following the Clinical and Laboratory Standards Institute (CLSI) rules. TLDSJ, TLDM, and TLDCH displayed a strong DPPH scavenging activity (EC_50_ = 42, 41.6, and 43 µg/mL, respectively) and inhibition of lipoperoxidation in erythrocytes (86–88% at 250 µg TLD/mL), while a less effect in the FRAP and TEACantioxidant assays was found. Additionally, the decoctions showed a content of phenolics compounds of 94 mg gallic acid equivalents (GAE)/g, 185 GAE/g, and 64 GAE/g, for TLDSJ, TLDM, and TLDCH samples, respectively. Regarding the flavonoid content, the Chilean sample was highlighted with 19 mg quercetin equivalents (QE)/g. In this work, several phenolic compounds, including sesquiterpenes, flavonoids, and phenolic acids, were rapidly identified in TLDSJ, TLDM, and TLDCH extracts by means UHPLC–PDA–OT-MS/MS for the first time, which gave a first scientific support to consider this medicinal decoction from both countries as a valuable source of metabolites with antioxidant effects, some with outstanding potential to improve human health.

## 1. Introduction

The complementary and alternative medicine consisting of the use of extracts, decoctions, infusions, waxes, exudates, tinctures, creams, emulsions, and propolis obtained from local medicinal plants is either the mainstay of healthcare delivery in developing countries or serves as a complement to standard medical care [1]. Secondary natural products from plants have, in the last century, become important compound leads for the development of new drug candidates for rational clinical therapy, exhibiting a variety of biological activities in experimental pharmacology, and serving as structural templates in medicinal chemistry. The exploration of plants and the discovery of natural compounds based on ethno-pharmacology are very important in the search of potential drug leads [2]. In addition, the ethno-botanical research on medicinal plants has assumed importance in the last decade, in response to the rapid loss of traditional knowledge, the decrease of plant diversity due to degradation of natural areas, as well as in the rise of prospecting plant-derived compounds. Furthermore, it is widely accepted that the re-activation of traditional knowledge about plants and associated management practices in a community is a tool that can contribute to development in those socially and economically depressed countries [3].

Several Andean medicinal species growing in Argentina and Chile are collected annually during their flowering season by Andean settlers, who transfer their knowledge and dried plants to herbalist collection centers or popular markets, located in large cities with high demands for these medicines [4,5].

On the other hand, interest in endemic Andean medicinal plants is high, as they represent a big source of potential novel biologically/pharmacologically active extracts or compounds, which are very little explored, that can offer potential candidates for the study of new drugs, and preparation of functional foods and food additives. One of the highlighted endemic Andean species is *Tessaria absinthioides* (Hook. & Arn.) DC. (Asteraceae), whose vernacular name is “pájaro bobo” in Argentina and “Brea” in Chile, which is used in the form of infusion or decoctions in traditional medicine as an hypocholesterolemic, balsamic, and expectorant to treat renal insufficiency, diabetes, and digestive disorders [6,7,8,9,10,11,12,13]. Previous studies related to its ethnopharmacological use have shown that *Tessaria absinthioides* possesses insecticidal, repellent, cytoprotective, viricidal, anti-inflammatory, cytotoxic, and antitumoral properties [10,11,12,13,14,15,16]. Regarding its chemical composition, mainly sesquiterpenes, sesquiterpenes sulphates, caffeoylquinic acids, other phenolics, and flavonoids have been previously isolated or identified from its aerial parts [6,15].

To our knowledge, there are no reports on chemical characterization and biological studies of decoctions obtained from this plant, which is the usual way of consumption in traditional medicine in South America. The search for polyphenols antioxidants or free radical scavengers in medicinal plants and other sources has been increasing, sustained mainly by the vital and beneficial role that these chemical compounds play in the human diet in the prevention of diseases or pathologies associated with stress oxidative and in maintaining good human health. High-resolution mass spectrometry is an outstanding and accurate technology to determine the presence of biologically active compounds in medicinal plants. In the last decade, extracts, decoctions, and infusions of medicinal plants and fruits native to Argentina and Chile have been analyzed using quadrupole Orbitrap spectrometry (Q-OT-MS), updating their polyphenols compositions significantly [17,18,19,20,21,22]. 

The main goals and novelty of this work are the antioxidant and antibacterial effects complemented with the full metabolic polyphenolic profile using a hybrid high-resolution mass spectrometer of the lyophilized decoctions from the medicinal plant *Tessaria absinthiodes*, to support the reputed medicinal properties of this plant.

## 2. Materials and Methods

### 2.1. Chemicals

Ultra-pure water (<5 µg/L TOC, (total organic carbon) was obtained from a water purification system Arium 126 61316-RO, plus an Arium 611 UV unit (Sartorius, Goettingen, Germany). Methanol (HPLC grade) and formic acid (puriss. p.a. for mass spectrometry) from J. T. Baker (Phillipsburg, NJ, USA) were obtained. Chloroform (HPLC grade) were from Merck (Santiago, Chile). Commercial Folin–Ciocalteu (FC) reagent, 2,2-diphenyl-1-picrylhydrazyl (DPPH), ferric chloride hexahydrate, 2,4,6-tris(2-pyridyl)-s-triazine, trolox, quercetin, gallic acid, DMSO, and HPLC standards (citric acid, vanillic acid, and chlorogenic acid, all standards with purity higher than 95% by HPLC) were purchased from Sigma-Aldrich Chem. Co. (St Louis, MO, USA) or Extrasynthèse (Genay, France). Cefotaxime was from Argentia^®^ (Bristol-Myers Squibb, Buenos Aires, Argentina). Mueller–Hinton broth was provided by Laboratorio Britania (Buenos Aires, Argentina).

### 2.2. Plant Material

A first sample of *T. absinthioides* from San Juan Province Argentina was collected in the locality “Medano de Oro”, department of Rawson, in December 2014. A voucher specimen was deposited in the Laboratory of Natural Products of the University of San Juan (Argentina) (voucher number IBT-TA-1). A second sample of *T. absinthioides*, from Mendoza province, was collected in Lavalle-County, Mendoza-Argentina (33°44′10″ S, 68°21′30.5″ W) in December 2014. A voucher specimen (voucher number MERL-61823) was deposited in the Mendoza Ruiz Leal herbarium. A third sample of *T. absinthioides* from Northern Chile was collected in El Tatio (Chile) in December 2014 (22°34′26.7″ S, 68°01′24.4″ W). A voucher herbarium specimen was deposited in the Laboratory of Natural products of the University of Antofagasta (Chile) (voucher number TA161114). All three aerial parts were collected, dried at room temperature, stored in the absence of light and heat, and then ground to prepare the decoctions. Samples were authenticated by the botanist Alicia Marticorena, University of Concepcion, Chile.

### 2.3. Lyophilized Decoction Preparation of T. absinthiodes (TLD)

Decoctions of *T. absinthiodes* from the Antofagasta, Mendoza, and San Juan collections were prepared at 10% weight/volume, from 100 g of dried and milled plant (leaves), in 1 L. of purified water by means of a PSA equipment. After 30 min of boiling, the decoctions were filtered, cooled for 24 h in a freezer at −40 °C, and then three representative samples of 100 mL of each decoction were subsequently lyophilized in an LA-B3 RIFICOR equipment (Buenos Aires, Argentina), obtaining the following yields: TLDCH: 2.0 ± 0.02%; TLDM: 2.40 ± 0.01%; and TLDSJ: 1.30 ± 0.01% w/v. The samples from the three locations were stored in a freezer at −40 °C until its use in the antioxidant assays, phenolics and flavonoids quantification, as well as in UHPLC–PDA–OT-MS analysis.

### 2.4. UHPLC–DAD–MS Instrument

An UHPLC-high-resolution MS machine Thermo Dionex Ultimate 3000 system with PDA detector controlled by Chromeleon 7.2 software (Thermo Fisher Scientific, Waltham, MA, USA) hyphenated with a Thermo Q-Exactive MS focus (Thermo, Bremen, Germany) was used [22]. For the analysis, 5 mg of the lyophilized material was dissolved in 2 mL of methanol, filtered through a 200-µm PTFE (polytetrafluoroethylene) filter, and 10 µL was injected in the instrument, considering all specifications as reported [22,23]. 

### 2.5. LC Parameters and MS Parameters

Liquid chromatography was performed using an UHPLC C18 column (Acclaim, 150 × 4.6 mm ID, 2.5 µm; Thermo Fisher Scientific, Bremen, Germany) operated at 25 °C. The detection wavelengths were 280, 254, 330, and 354 nm, and photodiode array detectors were set from 200 to 800 nm. Mobile phases were 1% formic aqueous solution (A) and acetonitrile 1% formic acid (B). The gradient program started at 5% B at zero time, then maintained 5% B for 5 min, then going to 30% B for 10 min, then maintaining 30% B for 15 min, then going to 70% B for 5 min, then maintaining 70% B for 10 min, and finally coming back to initial conditions in 10 and 12 min for column equilibration before each injection. The flow rate was 1.00 mL/min, and the injection volume was 10 µL. Standards and the lyophilized decoction dissolved in methanol were kept at 10 °C during storage in the autosampler. The HESI II and Orbitrap spectrometer parameters were optimized as previously reported [22,23]. Briefly, as follows: Sheath gas flow rate, 75 units; auxiliary gas unit flow rate, 20; capillary temperature, 400 °C; auxiliary gas heater temperature, 500 °C; spray voltage, 2500 V (for ESI−); and S lens, RF level 30. Full scan data in positive and negative were acquired at a resolving power of 70,000 FWHM at m/z 200. Scan range of m/z 100–1000; automatic gain control (AGC) was set at 3 × 10^6^ and the injection time was set to 200 ms. The chromatographic system was coupled to MS with a source II heated electro-nebulization ionization probe (HESI II). Nitrogen gas carrier (purity >99.999%) was obtained from a Genius NM32LA (Peak Scientific, Billerica, MA, USA) generator and used as a collision and damping gas. The mass calibration for Orbitrap was performed every day, in order to ensure the accuracy of an operating mass equal to 5 ppm. A mixture of taurocholic acid sodium salt, buspyrone hydrochloride, and sodium dodecil sulfate (Sigma-Aldrich, Darmstadt, Germany), plus Ultramark 1621 (Alpha Aezar, Stevensville, MI, USA), a fluorinated phosphazine solution, was used as standard mixture. These compounds were dissolved in a mixture of acetic acid, acetonitrile, water, and methanol (Merck, Santiago, Chile), and infused using a Chemyx Fusion 100 (Thermo Fisher Scientific, Bremen, Germany) syringe pump every day. The Q-Exactive 2.0 SP 2, Xcalibur 2.3 and Trace Finder 3.2 (Thermo Fisher Scientific, Bremen, Germany) were used for UHPLC mass spectrometer control and data processing, respectively.

### 2.6. Determination of Total Phenolic (TP) and Flavonoid (F) Content

The spectrophotometric quantification of total phenolic and flavonoid content was carried out following the previously reported methodology of Folin–Ciocalteu and AlCl*_3_* in microplate [20,21]. The TLDCH, TLDM, and TLDSJ were tested at 1 mg/mL using calibration curves of gallic acid and quercetin for the determination of values. The total phenolic was expressed as milligrams of gallic acid equivalents (GAE) per gram of extracts (mg GAE/g lyophilized decoction). Flavonoids were expressed as milligrams of quercetin equivalents (QE) per gram of extracts on (mg QE/g lyophilized decoction). The determinations were made in triplicate using a Multiskan FC Microplate Photometer (Thermo Scientific, Waltham, MA, USA). The values from triplicates are reported as the mean ± standard deviation (SD).

### 2.7. Antioxidant Activity

#### 2.7.1. DPPH Scavenging Activity

The potential of TLD as free radical scavenger was determined using the DPPH assay in microplate [20,21]. TLDCH, TLDM, TLDSJ, and quercetin (reference compound) dissolved in methanol were tested at concentrations between 1 and 100 µg/mL. The determinations were made in triplicate using a Multiskan FC Microplate Photometer (Thermo Scientific) and the EC_50_ concentration showing 50% of radicals scavenging activity was determined. The values are reported as the mean ± SD.

#### 2.7.2. Ferric-Reducing Antioxidant Power Assay (FRAP)

The spectrophotometric FRAP assay was carried out in microplate following the previously reported methodology [20,21,24]. Briefly, FRAP and TDLCH, TDLM, and TDLSJ dissolved in methanol to a concentration of 1 mg/ml were mixed and, on the other hand, a calibration curve was prepared by mixing FRAP and Trolox solutions, the latter at concentrations between 0 and 1 mmol /L. The absorbance determinations were made in triplicate using a Multiskan FC Microplate Photometer (Thermo Scientific). Results were obtained by linear regression from the FRAP–Trolox calibration plot and are shown in equivalent milligrams Trolox/g TLD.

#### 2.7.3. Trolox Equivalent Antioxidant Activity (TEAC) Assay

TEAC assay was carried out in microplate following the previously reported methodology [25,26]. Briefly, the TLDCH, TLDM, and TLDSJ were dissolved in methanol and mixed with 200 µL of ABTS, measuring the absorbance at 734 nm after 4 min. Results were obtained by linear regression from a calibration curve constructed with Trolox (reference compound, 0–1 mmol/L) and are expressed as equivalent milligrams Trolox/g TLD.

#### 2.7.4. Lipid Peroxidation in Human Erythrocytes

The lipid peroxidation in human erythrocytes (LP) assay was carried out following the previously reported methodology [20,21]. Briefly, human red blood cells obtained were washed successively in cold phosphate-buffered saline (PBS). After washing, the cells were suspended in PBS, regulating the density to 1 mM hemoglobin in each reaction tube. The final cell suspension was incubated in quadruplicate with different concentrations of 250 and 500 µg of TLD/mL and catechin (reference compound at 100 µg/mL). LP was induced with a solution of tert-Butyl hydroperoxide and spectrophotometrically quantified by the formation of TBARs. The results are expressed as a percentage of inhibition of LP.

### 2.8. Antibacterial Activity

Strains from the American Type Culture Collection (ATCC) American Type Culture Collection (ATCC), Rockville, MD, USA and clinical collections from Laboratorio de Microbiología, Hospital Marcial Quiroga, San Juan, Argentina (MQ) were used for this test. The microorganisms used were both Gram-positive and Gram-negative. Gram-positive: *Staphylococcus aureus* methicillin-sensitive ATCC 29213, *Staphylococcus aureus* methicillin-resistant ATCC 43300, clinical isolates of *Staphylococcus aureus* methicillin-resistant-MQ1, *Staphylococcus aureus* methicillin-resistant-MQ2, *Streptococcus agalactiae*-MQ3 and *Streptococcus pyogenes*-MQ4; and Gram-negative: *Escherichia coli* ATCC 25922. This activity was evaluated by means of the broth microdilution test, following the guidelines of the Clinical and Laboratory Standards Institute. The bacterium inoculum employed was 5 × 10^5^ CFU/mL. TLDs were dissolved in DMSO and tested at concentrations between 0.98 and 3000 µg/mL. Additionally, Cefotaxime (Argentia^®^, Buenos Aires, Argentina) was used as a positive control. After 24 h of incubation in microplates, the minimal inhibitory concentration (MIC) values were calculated from absorbances at 620 nm.

### 2.9. Statistical Analysis 

The results of the antioxidant assays were statistically treated to determine significant differences between their mean values, using InfoStat 26 (*p* < 0.05; Duncan’s test).

## 3. Results and discussion

### 3.1. UHPLC–OT Analysis of Tessaria Absinthiodes Decoctions from San Juan and Mendoza Provinces (Argentina) and Chile

The full scan mass spectra, base peak chromatograms, and data-dependent scan experiment were very useful for the identification of unknown phenolic compounds and sesquiterpenoid characteristics of this bioactive plant, since the Orbitrap provided high-resolution and accurate mass product ion spectra from precursor ions that were unknown beforehand within a single run. Combining full MS spectra and MS^n^ experiments, several compounds were tentatively identified in *T. absinthioides* decoction (TLD), including phenolic acids, two fatty acids, and several characteristic eudesmane sesquiterpenoids. Some of the compounds were identified by spiking experiments with available standards. As far as we know, some of the compounds are reported for the first time in this species. The generation of molecular formulas was performed using high resolution accurate mass analysis (HRAM) and matching with the isotopic pattern. In this work, only the negative mode of detection was used. Electrospray negative mode with an energy of “0” or “5” EV is the most abundantly used method to detect phenolics. Compounds with a phenolic OH easily lose the proton in electrospray ionization, giving very good and diagnostic parent ions and fragments.

**Figure 1:**
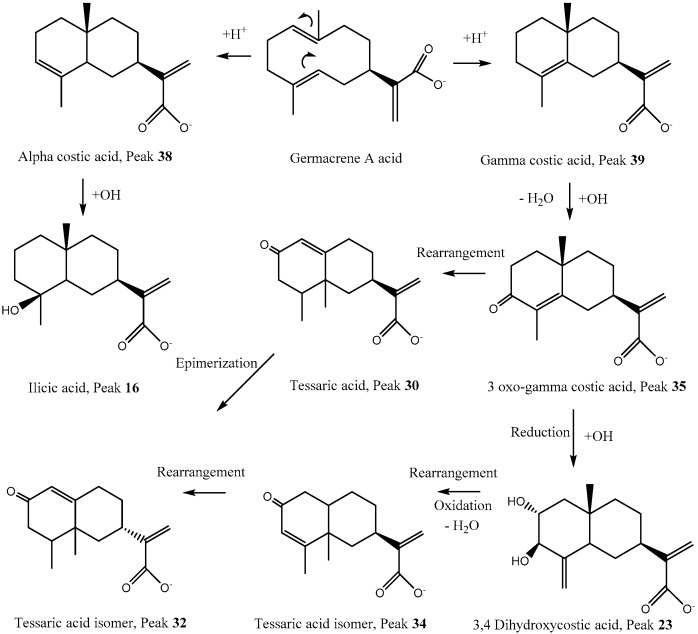
Proposed biosynthesis and structures of some eudesmane and eremophilane compounds detected in *T. absinthioides*.

**Figure 2:**
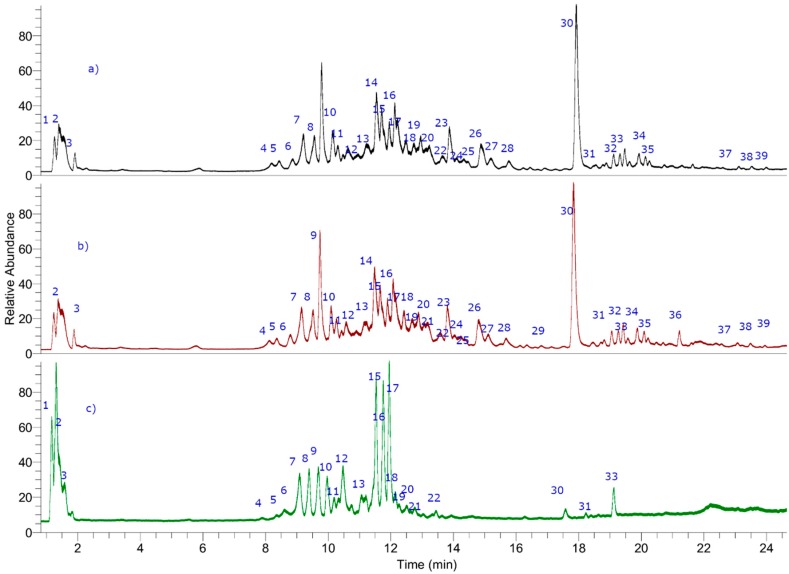
UHPLC–MS (total ion current) chromatograms of *T. absinthioides* lyophilized decoction from Argentina ((**a**) TLDSJ, San Juan sample and (**b)** TLDSM Mendoza sample) and Chile ((**c**) TLDCH Antofagasta sample).

Finally, analyses were confirmed using MS/MS data and comparing the fragments found with the literature. Below is the complete metabolome identification (Table 1 and Figure 1 and Figure 2, and Figure S1a–i, Supplementary Material).

#### 3.1.1. Phenolic Acids

Peak 6 with an [M−H]^−^ ion at *m/z*: 353.08671 was identified as chlorogenic acid or caffeoylquinic acid (CQA) (C_16_H_17_O_9_^−^) [22,23], peak 8 as vanillic acid (C_9_H_7_O_4_^−^), while peaks 14, 15, and 17 with pseudo-molecular ions around 515 Daltons and producing CQA MS^n^ ions around 353 Daltons (Table 1), and quinic acid daughter ions at m/z: 191 Daltons, were identified as isomers of di-CQA (1′,5′,3′,5′- and 4′,5′-di-CQA, respectively) [27]. In the same manner, peak 19 with an [M−H]^−^ ion at m/z: 677.15033 was identified as the anti-inflammatory compound 3′,4′,5′-tri-CQA (C_34_H_39_O_15_^−^) [28] (Figure S1c, Supplementary Material), peak 25 as ginnalin A (C_20_H_19_O_13_^−^), and peak 27 as tetra CQA (C_43_H_35_O_18_^−^) [29] (Figure S1e, Supplementary Material).

#### 3.1.2. Sesquiterpenes

Several compounds bearing the eudesmane skeleton were already reported as constituents of this plant [6,30]. Peak 9 (Figure S1a, Supplementary Material) was identified as the derivative 2, 5, 9-trihydroxy-3-O-arabinosyl-tessaric acid (C_20_H_31_O_10_^−^). Peak 11 was identified as hymenoxynin (C_21_H_34_O_9_^−^) [31]. Peak 16 with a pseudo-molecular ion at *m/z*: 251.16516 was identified as ilicic acid (C_15_H_23_O_3_^−^) (Figure 1) [6]. Peak 30, the main reported constituent of this plant, was assigned to tessaric acid (Figure 1 and Figure S1g, Supplementary Material) and its isomer compound peak 18 (Figure S1b, Supplementary Material) was assigned the structure eudesmane 4(15), 11(13)-dien-12, 5β-olide (C_15_H_19_O_3_^−^) [6]. Peak 28 was identified as the matricarin sesquiterpene phenolic compound scorzonerin (C_30_H_36_O_11_^−^) (Figure S1f, Supplementary Material) [32]. Other eudesmanes with the same formula were assigned as tessaric acid isomers (peaks 32 and 34), while peak 39 was identified as alpha costic acid and peak 38 as its isomer gamma costic acid (C_15_H_21_O_2_^−^) (Figure 1 and Figure S1i, Supplementary Material) fragments: 215.00955, (M−H_2_O) 205.15973 (M−CO), and peak 35 was identified as its derivative 3-oxo-gamma costic acid fragments: 219.13902 (M−CO), 231.13903 (M−H_2_O). Peaks 23 and 26 were identified as two of the isomers of 3,4 and 3,5-dihydroxy-costic acid, respectively ([6]; Figure S1d, Supplementary Material). Peak 33 with a [M−H]^−^ ion at *m/z*: 309.17090 (Figure S1h, Supplementary Material) was assigned as 5-acetyl, 3-hydroxy-4-dihydro-costic acid (C_17_H_25_O_5_^−^). The following Figure 1 shows a proposed biosynthesis and structures of some eudesmane and eremophilane compounds detected in *T. absinthioides* based on typical reactions in plants [33].

#### 3.1.3. Oxylipins or Fatty Acids

Two compounds were identified as polyhydroxylated unsaturated fatty acids known as the dietary antioxidants oxylipins [34,35]. Thus, peak 29 (ion at *m/z*: 327.21790) was identified as trihydroxy-octadecadienoic acid (C_18_H_31_O_5_^−^, fragment: 283.22787 (M−CO_2_)) [34] and peak 31 with an [M−H]^−^ ion at *m/z*: 329.23225 was assigned to trihydroxy-octadecaenoic acid (C_18_H_33_O_5_^–^, fragment: 285.24352 (M−CO_2_)) as previously reported by some of us from Keule fruits [34]. Finally, peak 7 was identified as the saturated diacid 3-hydroxyoctanedioic acid (3-hydroxysuberic acid, C_8_H_13_O_5_^−^) (Figure 2) [35].

#### 3.1.4. Other Compounds

Peak 1 was regarded as quinic acid, while peak 3 was regarded as citric acid (C_6_H_7_O_7_^−^), respectively, peak 2 as manoheptulose (C_7_H_13_O_7_^−^), peak 36 as eupatorine, while peaks 4, 5, 10, 12, 13, 20–22, 24, and 37 remain unknown (Figure 2).

### 3.2. Total Phenolic and Flavonoid Contents and Antioxidant and Antibacterial Activity

*Tessaria absinthioides* lyophilized decoctions were assessed in vitro for total content of phenolics and flavonoids, in addition to antioxidant properties (Table 2). The extracts TLDSJ, TLDM, and TLDCH displayed strong DPPH scavenging activity (EC_50_ between 42 and 43 µg/mL, respectively), as well as a good inhibition of lipoperoxidation in erythrocytes (between 86 and 88% at 250 µg TLD /mL). Regarding FRAP and ABTS antioxidant assays, the three decoctions showed a moderate effect in both trials. Argentinean decoctions showed a content of phenolic compounds of 94 and 185 mg GAE/g (TLDSJ and TLDM, respectively) and 64 mg GAE/g TLDCH. Regarding the flavonoid content, the Chilean sample was highlighted with 19 mg QE/g TLD. Concerning the content of phenolic compounds determined by the Folin–Ciocalteu method, TLDM extract showed twice as many phenolic compounds as compared to the other sample of Argentina (TLDSJ), and three times the content of the Chilean sample (TLDCH). However, TLDM and TLDCH have a similar flavonoid content.

The free radical scavenging activity shown by the decoctions could be associated with the presence of phenolic compounds capable of donating hydrogen in methanol solutions in which the test was carried out [36]. The antioxidant capacity detected is in concordance with the content of total phenolics in *Tessaria absinthiodes* decoction. On the other hand, in a cell-based model including human erythrocytes, lipid peroxidation was studied to evaluate the biological relevance of the antioxidant capacity of the decoction. The results showed that TLDSJ and TLDM prevented the hemolytic effect of the rupture of cell membranes induced by lipid peroxidation (86–86%, at 250 µg TLD/mL) compared with catechin (72% at 100 µg/mL). On the other hand, the TLDCH extract showed a stronger inhibition of the lipoperoxidation at 500 μg TLD/mL. The phenolic compounds contribute to the inhibition of the lipoperoxidation process by donating hydrogen atoms to the lipid radicals and producing lipid derivatives and antioxidant radicals, with greater stability, which are less available to promote autooxidation [37]. Recently, other Andean medicinal species with antioxidant potential have been reported with similar values of lipoperoxidation inhibition [20,21]. Phenolic compounds, ubiquitous in plants, are an essential part of the human diet and are of considerable interest due to their antioxidant properties and potential beneficial health effects. There is increasing evidence that the consumption of a variety of phenolic compounds in foods can reduce the risk of health disorders due to their antioxidant activity [38]. 

UHPLC–PDA–OT-MS profiles support that the decoctions of the medicinal plant *T. absinthiodes* from Argentina and Chile are a valuable natural resource of metabolites with antioxidant properties, either through their reducing capacities or their possible influence on intracellular redox states that improve human health in those ailments in which free radicals have proven participation, such as chronic inflammatory processes, or where contribute to development cancer by altering the phosphorylation state of multiple cell signaling pathways. Of the 30 detected metabolites, 22 compounds are reported for the first time. 

Citric acid, a natural compound present in a wide variety of fruits, acts as a synergist by promoting the activity of suitable antioxidants due to its metal chelating activity, which binds to heavy metals [39]. Studies conducted in rodents showed that citric acid protects liver tissue against the harmful effects of carbon tetrachloride (CCl_4_), lipopolysaccharide endotoxin (LPS), or the toxicity of organophosphorus insecticide malathion due to antioxidant action [40,41]. The depletion of proteins and glycogen in the liver of rats intoxicated with CCl_4_ is reduced by treatment with citric acid; additionally citric acid decreased the nitric oxide content, increased the activity of glutathione peroxidase (GPx), and decreased the expression of inducible nitric oxide synthase (iNOS) and caspase in the hepatic tissue of mice treated with LPS. Other assays have shown that citric acid decreased lipid peroxidation and increased GSH, and decreased the expression of iNOS in the liver of rats intoxicated with malathion. Furthermore, citric acid reduces the accumulation of nitric oxide in liver tissue by suppressing the activity of iNOS. It is likely that citric acid prevents liver damage through an antioxidant effect that limits the excessive generation of the EROs or nitrogen metabolites. Other authors have provided data suggesting an antioxidant action for citrate in the kidney [42]. In rat brain tissue, the antioxidant effect was reported on citrate and other intermediates of the tricarboxylic cycle [43]. In addition, Abdel-Salam et al. (2014) [44] etermined the decrease of lipid peroxidation (malondialdehyde) and nitrite by citric acid (1–4 g/kg) in the brain of mice treated with lipopolysaccharide b endotoxins.

Vanillic acid exerts protective effects in isoproterenol induced cardiotoxic rats due to its free radical scavenging, antioxidant, and anti-inflammatory properties [45]. Additionally, the modulator effect of vanillic acid on oxidant status in high fat diet-induced changes in diabetic hypertensive rats, involving reduction of blood glucose, insulin and blood pressure, combating oxidative stress by activation of tissue antioxidants has been recently reported [46]. On the other hand, eupatorin as a potential anticancer agent has been reported. The ability of eupatorin to non-specifically inhibit many protein kinases has already been proven and is the probable cause of the antiproliferative cellular effects published. Oncogenic kinases are vital proteins that couple extracellular signals with intracellular signaling pathways, which contribute to all stages of cancer development. Accumulated data reveal that plant compounds, particularly polyphenols, exert anticancer effects through acting on protein kinase signaling pathways [47]. The antioxidant, anti-inflammatory, hepatoprotective, antioxidant, hypocholesterolemic, and anti-apoptotic effects of caffeoylquinic acid were reported in several animal models [48,49,50,51].

Regarding di-CQA, Renzede et al. (2014) [52] have evaluated the antioxidant and antiherpes activity of 14 derivatives, including 3,5-dicaffeoylquinic acid (3,5-di-CQA). The isolated di-CQA derivatives showed similar activities for scavenging DPPH radicals, and inhibiting formation of cholesteryl ester hydroperoxide during copper ion induced rat blood plasma oxidation. In a previous study the di- CQA derivatives were evaluated, and showed similar activities to eliminate the DPPH radicals and inhibit the formation of cholesteryl ester hydroperoxide during the oxidation of the blood plasma of the rat induced by copper ions [53]. 

The neuroprotective properties of 3,5-di-CQA using a H_2_O_2_-induced apoptotic cell death model in SH-SY5Y human neuroblastoma cells were examined. The data suggest that 3,5-di-CQA might be a potential therapeutic agent for treating or preventing neurodegenerative diseases implicated with oxidative stress [54].

On the other hand, it is known that the sesquiterpenoid dehydrocostic acid inhibits leukotriene B4 production (IC_50_ = 22 µM), two kinds of pro-inflammatory enzymes: Elastase activity (IC_50_ = 43 µM) and bee venom phospholipase A_2_ activity (PLA_2_) (IC_50_ = 17 µM). Furthermore, this sesquiterpenoid was effective on some models of acute edema induced by PLA_2_ and 12-O-tetradecanoylphorbol 13-acetate (TPA) [55]. The anti-inflammatory action of many polyphenols is related to the inhibition of various enzymes involved in the metabolism of arachidonic acid, such as cyclooxygenase (COX), lipoxygenase (LO), nicotinamide adenine dinucleotide phosphate (NADPH) oxidase and xanthine oxidase (XO)·−, and the reduction of oxidative stress, through the capture of free radicals [56,57].

Recently, the phenolic compound ginnalin A displayed an apoptotic effect on human hepatocellular carcinoma cell line (Hep3B). Additionally, it showed a promising preventive activity against colon cancer cells (HCT116, SW480, and SW620) with IC_50_ values of 24.8 μM, 22.0 μM, and 39.7 μM, respectively [47,58].

The results of the antibacterial activity following the CLSI guidelines [59] are shown in Table 3. The TLDM extract showed a weak activity against Gram-positive bacteria, including *Staphylococcus aureus* methicillin-resistant ATCC 43300, *Staphylococcus aureus* methicillin-resistant-MQ-1, and *Staphylococcus aureus* methicillin-resistant-MQ-2 (MIC values between 2000 and 2500 µg/ml). The TLDCH and TLDM extracts showed no activity against most of the other strains tested (MIC values >2500 µg/mL). However, the TLDCH extract showed a weak activity against *Staphylococcus aureus* methicillin-resistant-MQ-2 (MIC = 2500 µg/mL). 

## 4. Conclusions

The potential antioxidant effect and chemical composition of TLD *Tessaria absinthioides* from three different collections were evaluated. We detected 39 peaks and determined 30 metabolites in the medicinal plant *T. absinthioides* (Figure 2), including 1 flavonoid (peak 36), 10 phenolic acids (peaks 1, 3, 6, 8, 14, 15, 17, 19, 25, and 27), 3 fatty acids (Peaks 7, 29, and 31), and 13 sesquiterpenes (peaks 9, 11, 18, 23, 26, 28, 30, 32–35, 38, and 39), which were identified in used decoctions for the first time. Among the 30 determined metabolites by full high resolution MS spectra and some diagnostic MS fragments, compounds 1, 3, 7–9, 14, 15, 17–19, 23, 25–29, 31–35, 38, and 39 are reported for the first time for this species. UHPLC–PDA–OT-MS profiles prove that the decoctions of the medicinal plant *T. absinthiodes* from Argentina and Chile are valuable natural resources supported by their antioxidant properties, either through their reducing capacities or their possible influence on intracellular redox states, that can help to improve human health in those ailments in which free radicals have proven participation, such as chronic inflammatory processes or where contribution to development cancer by altered phosphorylation states of multiple cell signaling pathways. Additionally, this research opens a pathway for the development of phytomedicine products from *T. absinthioides.*

## Figures and Tables

**Table 1:** High resolution UHPLC–PDA–Q-Orbitrap identification of metabolites from TLD.

Peak	Retention Time (min)	UV Max	Tentative Identification	Elemental Composition [M−H]	Measured Mass (m/z)	Theoretical Mass (m/z)	Ac Curacy (δppm)	MSn Ions (δppm)
1	1.21	-	Quinic acid *^,a,b,c^	C_7_H_11_O_6_^−^	191.05579	191.05501	4.03	144.00844
2	1.31	-	Manoheptulose ^a,b,c^	C_7_H_13_O_7_^−^	209.06633	209.06558	3.37	153.01857
3	1.82	-	Citric acid ^a,b,c^	C_6_H_7_O_7_^−^	191.01863	191.01939	3.76	144.00844
4	7.26	-	Unknown ^a,b,c^	C_16_H_11_O_15_N_3_^−^	365.01859	365.01847	0.23	-
5	8.47	330	Unknown ^a,b,c^	C_4_H_7_O_12_^−^	246.99167	246.99320	−6.2	152.01080
6	8.73	239–320	CQA (chlorogenic acid) * ^a,b,c^	C_16_H_17_O_9_^−^	353.08671	353.08786	3.86	275.0235, 191.05481 (quinic acid), 707.18115 (2M−H)
7	9.55	223	3-Hydroxysuberic acid ^a,b,c^	C_8_H_13_O_5_^−^	189.07645	189.07575	3.69	-
8	10.03	330	Vanillic acid * ^a,b,c^	C_9_H_7_O_4_^−^	179.03465	179.03389	4.28	135.04436
9	10.07	283	2, 5, 9-trihydroxy-3-O-arabinosyl-tessaric acid ^a,b,c^	C_20_H_31_O_10_^−^	431.19241	431.19117	2.87	311.11367, 135.04433
10	10.36	330	Unknown ^a,b,c^	C_10_H_9_O_15_N_3_^−^	411.00293	411.00282	0.32	-
11	10.43	288–346	Hymenoxynin ^a,b,c^	C_21_H_34_O_9_^−^	429.21313	429.21191	2.85	267.21184 (M−hexose moiety)
12	11.12	330	Unknown ^a,b,c^	C_15_H_7_O_11_N^−^	377.00092	377.00136	−1.18	
13	11.48	330	Unknown ^a,b^	C_10_H_9_O_15_N_3_^−^	411.00296	411.00282	0.34	
14	11.62	239–320	1′,5′-di-CQA(cynarin) ^a,b,c^	C_25_H_23_O_12_^−^	515.11945	515.11840	2.02	191.05551 (quinic acid), 179.03429
15	11.82	239–320	3′,5′-di-CQA ^a,b,c^	C_25_H_23_O_12_^−^	515.11951	515.11840	2.14	191.05562 (quinic acid), 179.03429
16	11.90	255–354	Ilicic acid ^a,b,c^	C_15_H_23_O_3_^−^	251.16516	251.16417	4.5	233.15470 (M–H_2_O), 207.17544 (M−CO_2_), 171.95076
17	12.00	239–320	4′,5′-di-CQA ^a,b,c^	C_25_H_23_O_12_^−^	515.11840	515.11945	2.14	191.05551 (quinic acid), 179.03429
18	12.21	255–365	Eudesmane 4(15), 11(13)-dien-12, 5βolide * ^a,b,c^	C_15_H_19_O_3_^−^	247.13380	247.13287	3.77	205.15968 (M−CO_2_), 149.09645
19	12.57	289–329	3′,4′,5′-tri-CQA ^a,b,c^	C_34_H_39_O_15_^−^	677.15033	677.15119	14.30	515.11963 (Di-CQA), 191.05561 (quinic acid)
20	12.81	278	Unknown ^a,b,c^	C_30_H_33_O_15_N_10_^−^	773.13715	773.21214	−0.08	
21	12.96	278	Unknown ^a,b,c^	C_26_H_8_ON_10_^−^	476.08734	476.08771	−0.76	
22	13.21	278	Unknown ^a,b,c^	C_12_HO_16_N_14_^−^	953.17645	953.17632	−0.98	476.08722
23	13.46	278	3,4-Dihydroxy-costic acid ^a,b^	C_15_H_21_O_4_^−^	265.14459	265.14344	4.32	247.13395 (M−H_2_O)
24	14.04	278	Unknown ^a,b^	C_28_H_23_O_12_N_9_^−^	677.15039	677.14741	4.40	
25	14.70	323	Ginnalin A ^a,b^	C_20_H_19_O_13_^−^	467.08200	467.08202	−0.03	249.08006
26	16.20	278	3,5-Dihydroxy-costic acid ^a,b^	C_15_H_21_O_4_^−^	265.14456	265.14344	4.24	247.13392 (M−H_2_O)
27	15.55	265–329	Tetra-CQA ^a,b^	C_43_H_35_O_18_^−^	839.17999	839.18289	3.43	191.05552 (quinic acid), 179.03423
28	15.94	235	Scorzonerin (C_30_H_36_O_11_^−^) ^a,b^	C_30_H_36_O_11_^−^	571.21872	571.21739	1.63	467.08185, 327.21765
29	16.48	222	Trihydroxy-octadecadienoic acid ^a^	C_18_H_31_O_5_^−^	327.21790	327.21660	3.95	283.22787(M−CO_2_)
30	17.69	283	Tessaric acid *^,a,b,c^	C_15_H_19_O_3_^−^	247.13379	247.13287	3.71	205.15979 (M−CO_2_), 149.09644
31	18.34	222	Trihydroxy-octadecaenoic acid ^a,b^	C_18_H_33_O_5_^−^	329.23225	329.23364	4.22	285.24352(M−CO_2_)
32	19.09	283	Tessaric acid isomer ^a,b^	C_15_H_19_O_3_^−^	247.13383	247.13287	3.90	205.15972 (M−CO_2_), 163.11223
33	19.25	218	5-Acetyl-3-hydroxy-4-dihydro-costic acid ^a,b,c^	C_17_H_25_O_5_^−^	309.17090	309.16965	4.03	291.16019 (M−H_2_O), 267.16018(M-acetyl moiety) 152.08374
34	19.47	283	Tessaric acid isomer ^a,b^	C_15_H_19_O_3_^−^	247.13383	247.13287	3.90	205.15979 (M−CO_2_), 162.01357
35	19.96	283	3-oxo-gamma costic acid ^a,b^	C_15_H_19_O_3_^−^	247.13383	247.13287	3.90	231.13903, 233.11812, 219.13902, 215.00955, 149.09644
36	21.53	218	Eupatorine ^a,b^	C_13_H_27_O_8_^−^	343.08258	343.08123	−4.11	329.06663 (M−CH_3_), 315.0533 (M−2CH_3_), 313.03531
37	22.53	218	Unknown ^a,b^	C_13_H_27_O_8_^−^	311.16876	311.17004	−4.11	
38	23.41	335	Gamma costic acid ^a,b^	C_15_H_21_O_2_^−^	233.15453	233.15361	3.94	215.00955, (M–H_2_O) 205.15973(M−CO)
39	23.97	335	Alpha Costic acid ^a,b^	C_15_H_21_O_2_^−^	233.15451	233.15361	3.87	215.00953, (M−H_2_O) 205.15965 (M−CO)

* Identified by spiking experiments with authentic standards. Identified in ^a^ TDLSJ; ^b^ TDLM; ^c^ TDLCH.

**Table 2:** The antioxidant and total phenolic and flavonoid content of TLD from Argentina and Chile. No significant differences were found between the three samples. ANOVA (analysis of variance) followed by Dunett’s comparison test was used (significance *p* ≤ 0.05).

Assay	TLDSJ	TLDM	TLDCH
Content of phenols			
Total phenolics (mg GAE/g TLD)	94.84 ± 12.76	185.53 ± 43.9	64.36 ± 5.53
Flavonoids (mg QE/g TLD)	9.10 ± 0.90	18.18 ± 5.3	19.16 ± 0.66
Antioxidant			
DPPH (EC_50_ in µg TLD/mL)	42.39 ± 8.21	41,6 ± 0.75	43.11 ± 4.04
FRAP (mM TE/g TLD)	0.89 ± 0.07	1.93 ± 0.3	0.98 ± 0.11
TEAC (mg TE/g TLD)	0.97 ± 0.04	1.29 ± 0.02	0.89 ± 0.085
Percentage LP (at 250 µg TLD/mL)	88.79 ± 11.53	86.77 ± 4.17	97.64 ± 1.9 ^a^

^a^ 500 μg/mL.

**Table 3:** Antimicrobial activity of *Tessaria absinthiodes.*

Antibacterial Assay	TLD (MICs in µg/mL)			
	TLDCH	TLDM	TLDSJ	Cefotaxime
*Staphylococcus aureus* methicillin-sensitive ATCC 29213	>2500	>2500	>2500	0.5
*Staphylococcus aureus*, methicillin-resistant ATCC 43300	>2500	2500	>2500	0.5
*Staphylococcus aureus* methicillin-resistant-MQ-1	>2500	2000	>2500	0.5
*Staphylococcus aureus* methicillin-resistant-MQ-2	2500	2000	>2500	0.75
*Streptococcus pyogenes-*1	>2500	>2500	>2500	0.25
*Escherichia coli* ATCC 25922	>2500	>2500	>2500	1.9

## Supplementary Materials

The following are available online at https://www.mdpi.com/2076-3921/8/12/593/s1: Figure S1 (**a**–**i**) Full Orbitrap MS spectra of compounds 9 (**a**), 18 (**b**), 19 (**c**), 23 (**d**), 27 (**e**), 28 (**f**), 30 (**g**), 33 (**h**), and 38 (**i**).
