# Supplementary material for: UHPLC–Q/Orbitrap/MS/MS Fingerprinting, Free Radical Scavenging, and Antimicrobial Activity of Tessaria absinthiodes (Hook. & Arn.) DC. (Asteraceae) Lyophilized Decoction from Argentina and Chile
Source: Antioxidants (Basel). 2019 Nov 28;8(12):593. doi: 10.3390/antiox8120593 (PMC6943634; doi:10.3390/antiox8120593)
Supplement: Supplementary file 1 [file antioxidants-08-00593-s001.pdf]

Supplementary Material for the article:

UHPLC-Q/Orbitrap/MS/MS fingerprinting, free radical scavenging and antimicrobial activity of *Tessaria absinthiodes* (Hook. & Arn.) DC. (Asteraceae) lyophilized decoction from Argentina and Chile.

Jessica Gómez [1,2], Mario J. Simirgiotis [3,4,*], Beatriz Lima [1,2], Carlos Gamarra-Luques [2,5,6], Jorge Bórquez [7], Duilio Caballero [8], Gabriela Egly Feresin [1,2] and Alejandro Tapia [1,*]

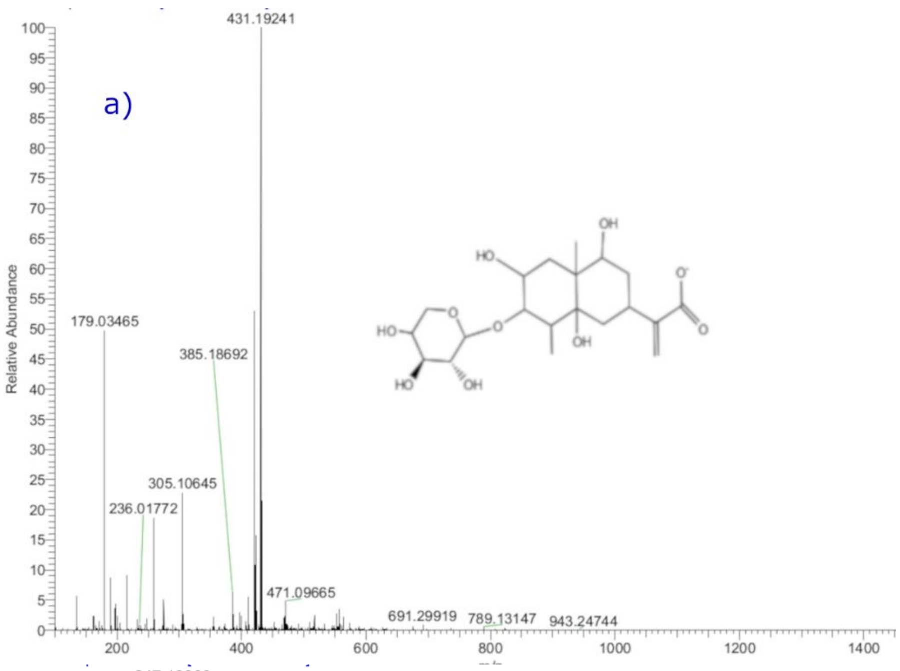
431.19241
a)
Relative Abundance
179.03465
385.18692
305.10645
236.01772
471.09665
691.29919
789.13147
943.24744

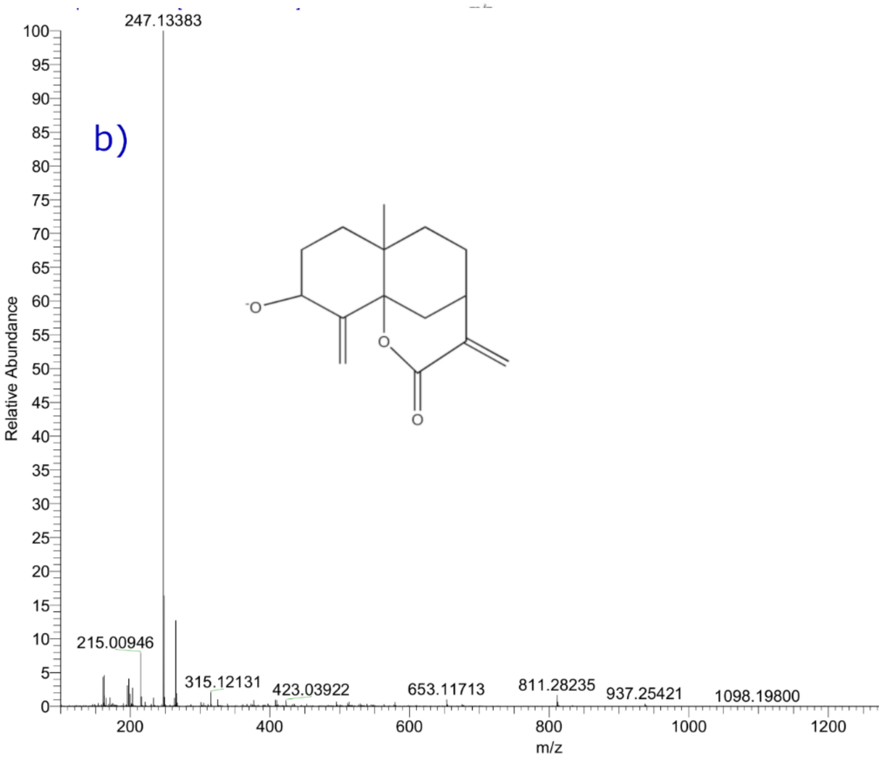
247.13383
b)
Relative Abundance
215.00946
315.12131
423.03922
653.11713
811.28235
937.25421
1098.19800
m/z

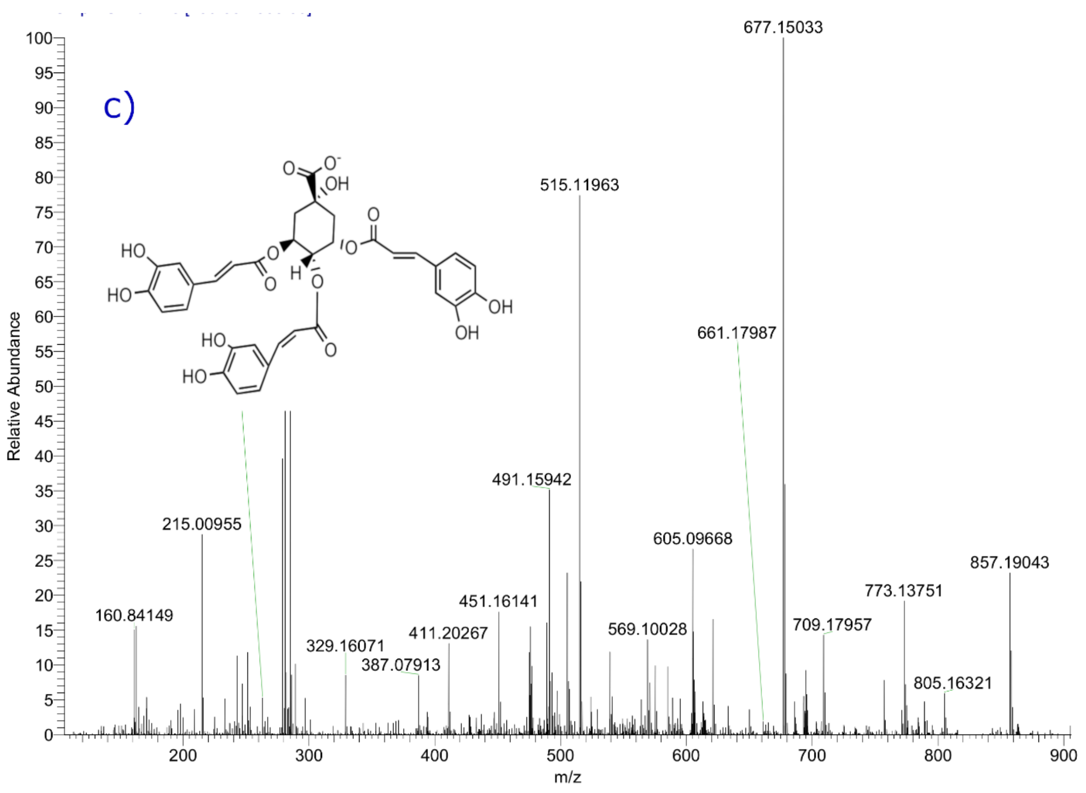
c)
677.15033
515.11963
661.17987
491.15942
215.00955
605.09668
857.19043
773.13751
451.16141
160.84149
569.10028
709.17957
411.20267
329.16071
387.07913
805.16321
Relative Abundance
m/z

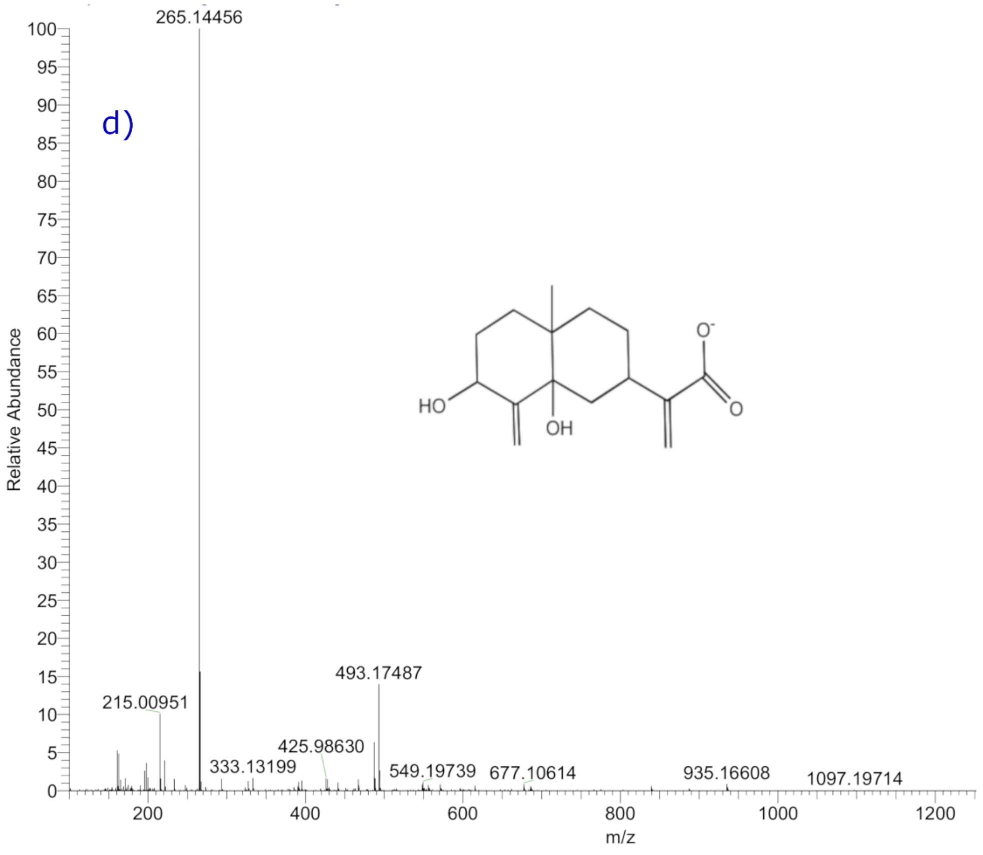
d)
265.14456
493.17487
215.00951
425.98630
333.13199
549.19739
677.10614
935.16608
1097.19714
Relative Abundance
m/z

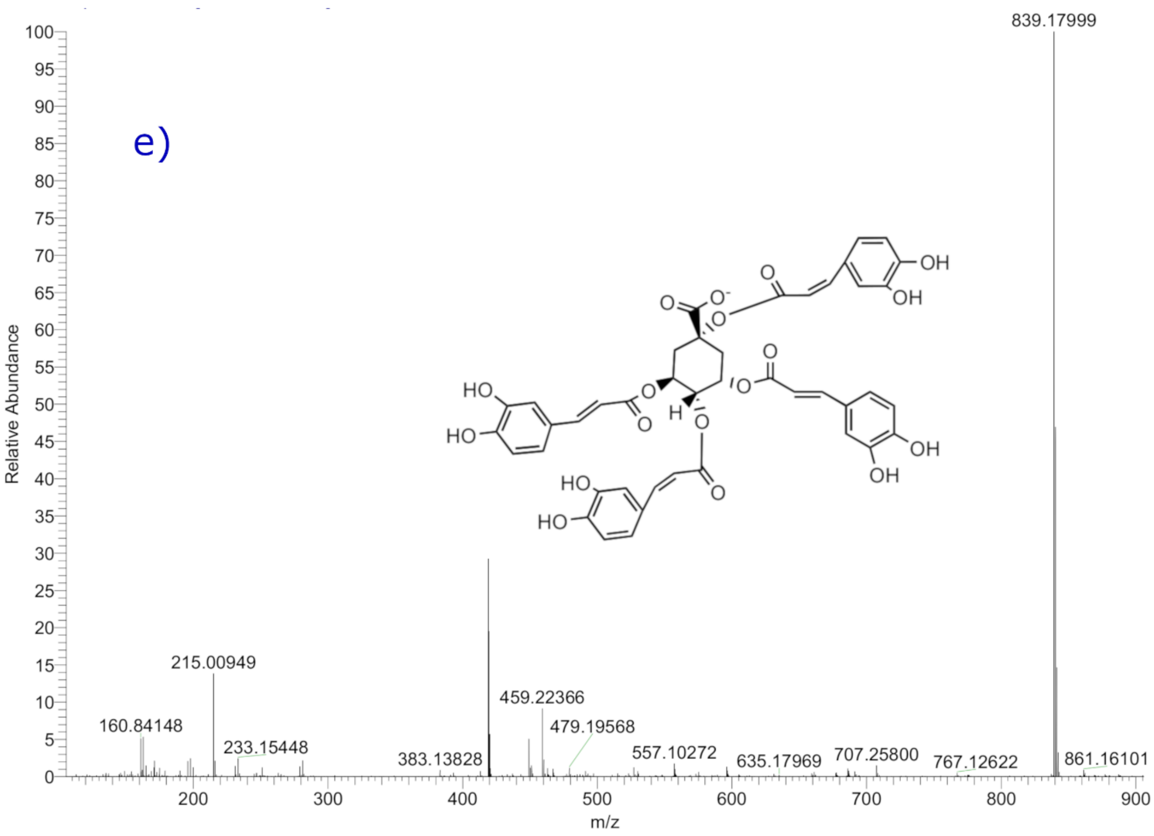

e)
839.17999
215.00949
459.22366
479.19568
160.84148
233.15448
383.13828
557.10272
635.17969
707.25800
767.12622
861.16101
Relative Abundance
m/z

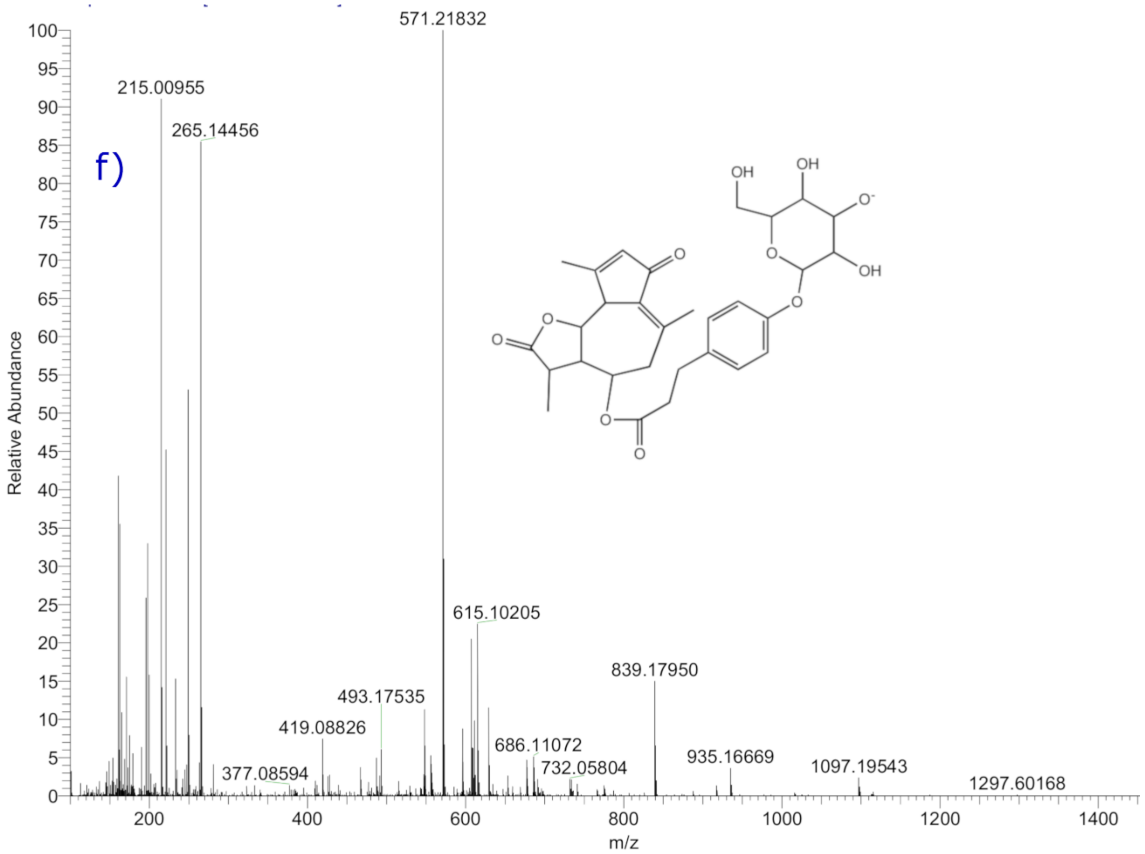

f)
571.21832
215.00955
265.14456
615.10205
839.17950
493.17535
419.08826
686.11072
732.05804
377.08594
935.16669
1097.19543
1297.60168
Relative Abundance
m/z

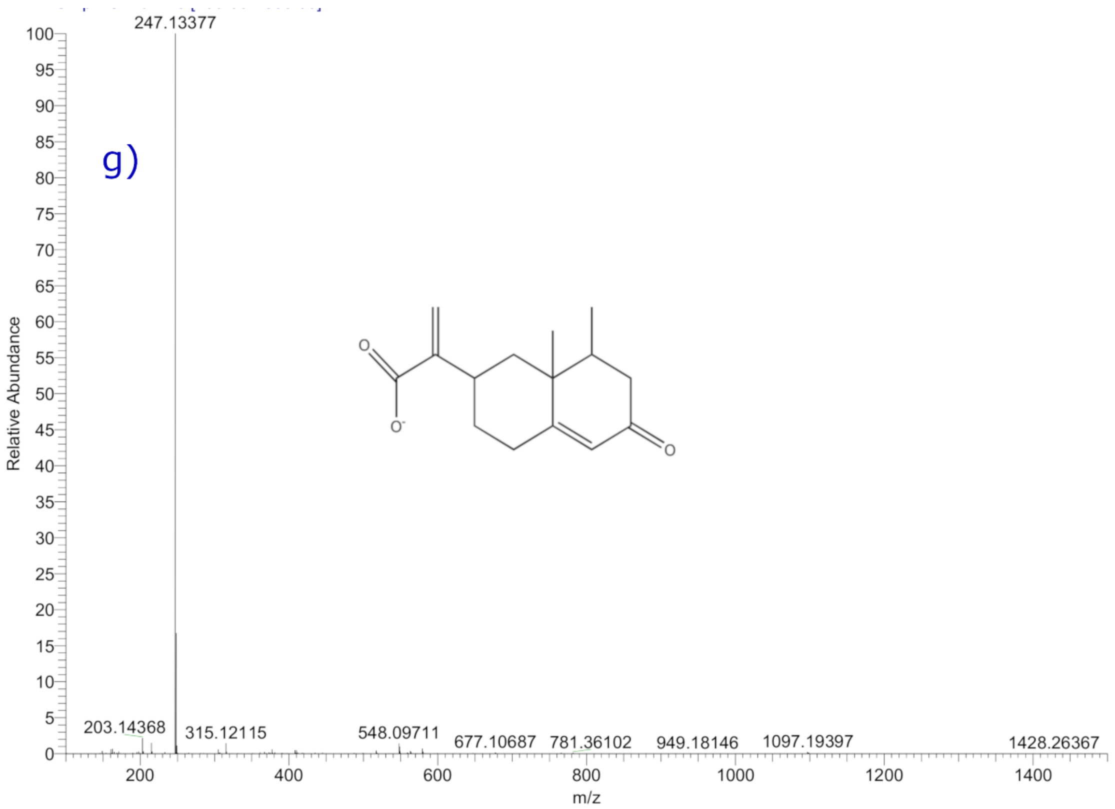

g)
247.13377
203.14368
315.12115
548.09711
677.10687
781.36102
949.18146
1097.19397
1428.26367
Relative Abundance
m/z

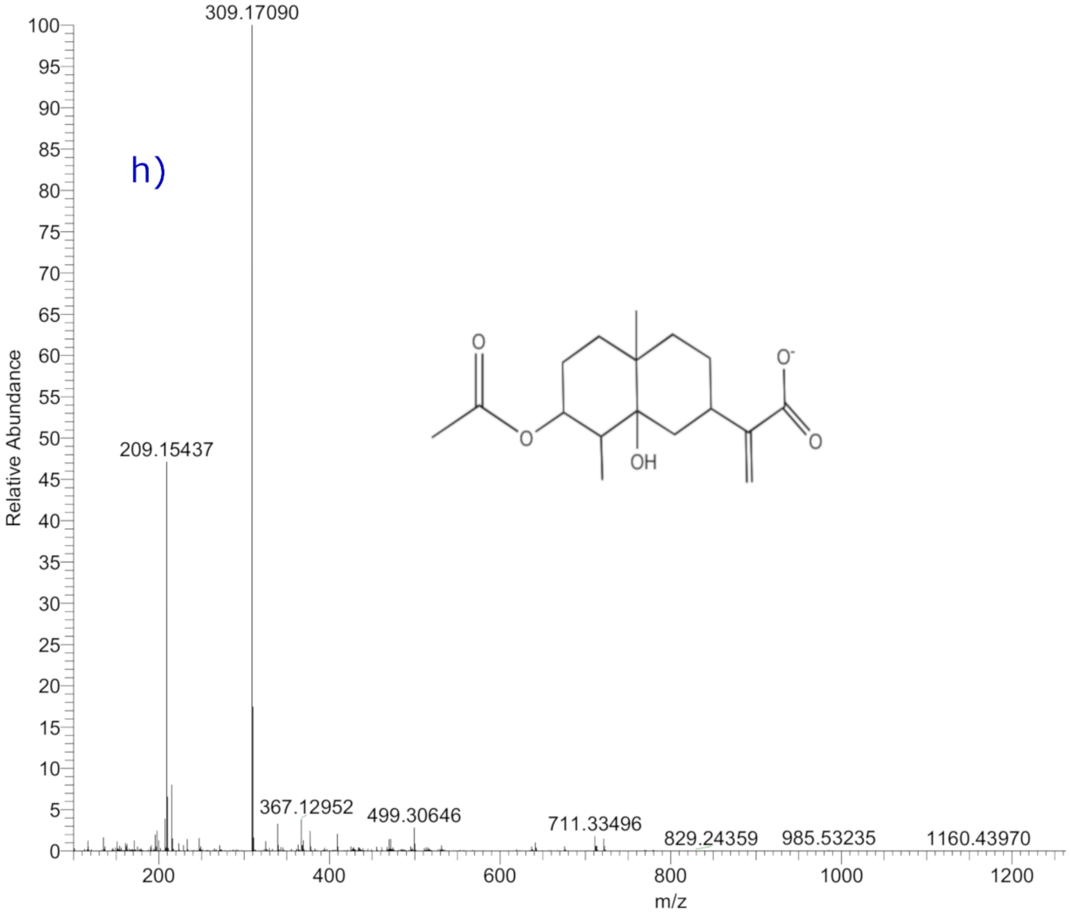

h)
309.17090
209.15437
367.12952
499.30646
711.33496
829.24359
985.53235
1160.43970
OH
Relative Abundance
m/z

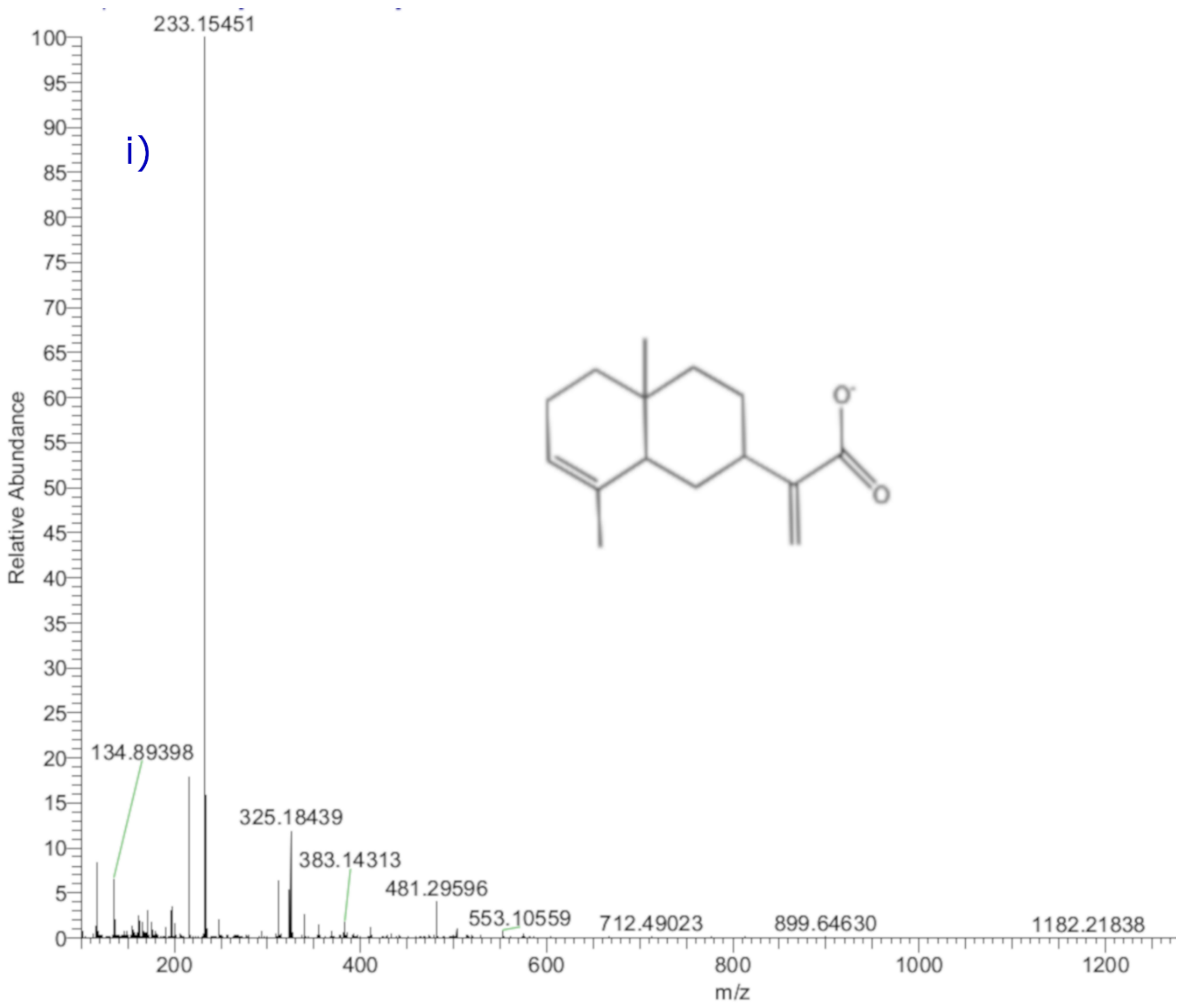

Figure S1. (**a–i**) Full orbitrap MS spectra of compounds 9(**a**), 18(**b**), 19(**c**), 23(**d**), 27(**e**), 28(**f**), 30(**g**), 33(**h**), and 38(**i**).
